# Human breast cancer cells educate macrophages toward the M2 activation status

**DOI:** 10.1186/s13058-015-0621-0

**Published:** 2015-08-05

**Authors:** Sofia Sousa, Régis Brion, Minnamaija Lintunen, Pauliina Kronqvist, Jouko Sandholm, Jukka Mönkkönen, Pirkko-Liisa Kellokumpu-Lehtinen, Susanna Lauttia, Olli Tynninen, Heikki Joensuu, Dominique Heymann, Jorma A. Määttä

**Affiliations:** School of Pharmacy, Faculty of Health Sciences, University of Eastern Finland, Yliopistonranta 1C, P.O. Box 1627, FI-70211 Kuopio, Finland; INSERM, UMR957, Equipe LIGUE 2012, Nantes, F-44035 France; Université de Nantes, Nantes atlantique universités, Laboratoire de Physiopathologie de la Résorption Osseuse et Thérapie des Tumeurs Osseuses Primitives, Nantes, F-44035 France; CHU de Nantes, Nantes, F-44035 France; Institute of Biomedicine, Department of Cell Biology and Anatomy, University of Turku, Turku, Finland; Cell Imaging Core, Turku Centre for Biotechnology, University of Turku, and Åbo Akademi University, Turku, Finland; Medical School, University of Tampere and Department of Oncology Tampere University Hospital, Tampere, Finland; Laboratory of Molecular Oncology, Biomedicum Helsinki, University of Helsinki, Helsinki, Finland; Department of Pathology, Haartman Institute, University of Helsinki and HUSLAB, Helsinki, Finland; Comprehensive Cancer Center, Helsinki University Hospital, and Department of Oncology, University of Helsinki, Helsinki, Finland

## Abstract

**Introduction:**

The immune system plays a major role in cancer progression. In solid tumors, 5-40 % of the tumor mass consists of tumor-associated macrophages (TAMs) and there is usually a correlation between the number of TAMs and poor prognosis, depending on the tumor type. TAMs usually resemble M2 macrophages. Unlike M1-macrophages which have pro-inflammatory and anti-cancer functions, M2-macrophages are immunosuppressive, contribute to the matrix-remodeling, and hence favor tumor growth. The role of TAMs is not fully understood in breast cancer progression.

**Methods:**

Macrophage infiltration (CD68) and activation status (HLA-DRIIα, CD163) were evaluated in a large cohort of human primary breast tumors (562 tissue microarray samples), by immunohistochemistry and scored by automated image analysis algorithms. Survival between groups was compared using the Kaplan-Meier life-table method and a Cox multivariate proportional hazards model. Macrophage education by breast cancer cells was assessed by *ex vivo* differentiation of peripheral blood mononuclear cells (PBMCs) in the presence or absence of breast cancer cell conditioned media (MDA-MB231, MCF-7 or T47D cell lines) and M1 or M2 inducing cytokines (respectively IFN-γ, IL-4 and IL-10). Obtained macrophages were analyzed by flow cytometry (CD14, CD16, CD64, CD86, CD200R and CD163), ELISA (IL-6, IL-8, IL-10, monocyte colony stimulating factor M-CSF) and zymography (matrix metalloproteinase 9, MMP-9).

**Results:**

Clinically, we found that high numbers of CD163^+^ M2-macrophages were strongly associated with fast proliferation, poor differentiation, estrogen receptor negativity and histological ductal type (*p*<0.001) in the studied cohort of human primary breast tumors. We demonstrated *ex vivo* that breast cancer cell-secreted factors modulate macrophage differentiation toward the M2 phenotype. Furthermore, the more aggressive mesenchymal-like cell line MDA-MB231, which secretes high levels of M-CSF, skews macrophages toward the more immunosuppressive M2c subtype.

**Conclusions:**

This study demonstrates that human breast cancer cells influence macrophage differentiation and that TAM differentiation status correlates with recurrence free survival, thus further emphasizing that TAMs can similarly affect therapy efficacy and patient outcome.

**Electronic supplementary material:**

The online version of this article (doi:10.1186/s13058-015-0621-0) contains supplementary material, which is available to authorized users.

## Introduction

Metastasis is often explained with the ‘seed and soil’ theory. Conceptually, it implies that the cancer cell (seed) undergoes epithelial to mesenchymal transition (EMT), invades vessels, becomes a circulating tumor cell (CTC), migrates, extravasates, undergoes mesenchymal to epithelial transition, and eventually colonizes distant sites as a disseminating tumor cell (DTC). ‘Soil’ relates to tumor microenvironment elements which contribute to these processes, making the distant sites permissive to colonization by CTCs or DTCs [1].

The immune system is a major player in the cancer cell/tumor microenvironment crosstalk. In solid tumors, 5−40 % of the tumor mass consists of tumor-associated macrophages (TAMs). Approximately 80 % of the publications in this field report an association between TAMs and poor prognosis [2, 3]. In humans macrophage polarization is a continuum that spans two extremes from the classically activated M1 macrophages to the alternatively activated M2 macrophages. M1 macrophages derive from interferon γ (IFN-γ) or lipopolysaccharide (LPS) stimuli and secrete inflammatory cytokines (e.g., IL-6, IL-12, reactive oxygen species (ROS), reactive nitrogen species (RN) and TNF-α). The validated surface-markers of human M1 macrophages include high levels of CD14 and CD16, CD64, CD86 and HLA-DRα [4, 5]. M2 macrophages, can be further divided into M2a, M2b and M2c macrophages. M2a macrophages arise from IL-4 or IL-13 stimuli and release matrix-remodeling cytokines. Elevated expression of CD200R and CD86 is a validated phenotypic marker of M2a macrophages [4, 5]. M2b macrophages result from the recognition of immune complexes in combination with IL-1β or LPS stimuli and like M2a macrophages, they are involved in wound healing. The immunosuppressive M2c-macrophages are the outcome of IL-10, TGF-β (transforming growth factor β), glucocorticoids or immune complex rich environments. M2c macrophages generate further IL-10 and matrix-remodeling factors such as matrix metalloproteinases (MMPs) [4, 5]. Elevated CD163 expression is a validated marker of M2c polarization [5].

TAMs, a macrophage population recruited and educated by tumor cells, which are therefore exposed to IL-10, TGF-β, M-CSF (monocyte colony stimulating factor) [6] and other immunosuppressive stimuli [7], are more closely related to the M2 type [8]. In the tumor microenvironment, TAMs will preferentially perform trophic and immunosuppressive rather than immune effector tasks [3, 9, 10]. Hence, TAMs promote epithelial outgrowth and invasion, which are common features of development and cancer [3, 9]. Wickoff et al. have shown that mammary tumors exhibit a paracrine loop between TAMs and cancer cells. TAMs express monocyte colony stimulating factor receptor (M-CSFR, also known as CSF-1R or cFMS), which binds monocyte colony stimulating factor (M-CSF, also known as CSF-1) secreted by cancer cells. Conversely, TAMs secrete epidermal growth factor (EGF) and activate the EGF receptor (EGFR) on the cancer cells. This allows co-migration of the two cell types, thus, enhancing motility and subsequent invasion of healthy surrounding tissue and intravasation [11, 12]. Also, breast cancer cell leucocyte receptor, vascular cell adhesion molecule 1 (VCAM1) binding to TAM α4-integrin explains the increased survival of VCAM1^+^ tumor cells in leucocyte-rich environments [13].

Like their phenotype and interactions with tumor cells, the location of TAMs in relation to hypoxic areas is a key parameter controlling tumor growth. In addition to the perivascular TAMs, which take part in cancer cell invasion [11, 12], TAMs are also recruited into hypoxic areas [14]. Within these avascular areas TAMs alter their gene expression profile, favoring a pro-tumor M2 phenotype [15]. This may explain why in the early stages [16] of cancers of the lung [17], colon [18] and stomach [19], the macrophages in the normoxic milieu display an M1 phenotype and are associated with good prognosis.

Immunohistopathological breast carcinoma studies with restricted numbers of samples (n = 53 and 120, respectively) reveal a gradual increase in the amount of infiltrating macrophages (CD68^+^) from normal breast tissue to benign proliferative breast disease, ductal carcinoma in situ (DCIS) and infiltrating ductal carcinoma [20, 21]. Two larger studies (n = 1,322 and 168, respectively) confirmed that CD68^+^ macrophages were associated with higher tumor grade, estrogen receptor (ER) and progesterone receptor (PR) negativity, human epithelial growth factor receptor 2 (HER-2) positivity and a basal phenotype, but led to the conclusion that CD68 expression was not an independent prognostic factor [22, 23]. Another breast cancer cohort study (n = 144), looking at total macrophage number (CD68^+^) and M2 macrophages (CD163^+^) found that CD163 was also associated with other prognostic markers [24]. It showed that CD68^+^ cells in the tumor stroma but not in the tumor nest were an independent prognostic factor for decreased cancer-specific survival, accounting for the localization of TAMs in the tumors more than their mere presence. Triple-negative/basal-like breast tumor stroma had more CD163^+^ and CD68^+^ cells and a higher proportion of CD163 relative to CD68 when compared to the stroma of luminal A tumors. This indicates a predominance of mature M2 macrophages and possibly immature myeloid-derived cells (MDCs, also CD163^+^) in triple-negative disease [24].

Several clinical studies have found an association between macrophage infiltration and angiogenesis in breast cancer [22, 25–28]. However, in relation to prognosis it is unanimous that larger studies of macrophage subpopulations are needed. This study intends to fill that gap. Focusing on the expression of M1 and M2 markers in samples from a large cohort of patients with breast cancer (n = 562), we looked for possible associations with tumor progression. Additionally, by studying the ex vivo differentiation of human macrophages in the presence of breast cancer conditioned media (CM), we aimed to find possible mechanisms of TAM education. To achieve these aims, we revisited tissue microarrays from a large cohort [29] of early human breast tumors of different subtypes, grades and aggressiveness and used different breast cancer cell lines.

## Methods

### Human samples

TMA samples (n = 562 out of 1,199 patients from the FinXX study, NCT00114816 [29]) were studied retrospectively. Clinicopathological characteristics of the sub-cohort are described in Table 1. Formalin-fixed, paraffin-embedded tumor samples were used for TMA. Blocks were made using a 1.0-mm tissue cylinder through a histologically representative area of each donor tumor block. From each donor block, 2–4 cores were cut and 15 TMA blocks were prepared, each containing 61–84 tumor samples plus 2–3 liver samples as positive controls.

**Table 1:** Patient demographics and relevant clinical characteristics

Factor	Entire series	CD68^a^		*P* ^b^	CD163^a^		*P* ^b^	HLA-Drα^a^		*P* ^b^
		≤369	>369		≤167.5	>167.5		≤107	>107	
	n = 562	n = 277	n = 274		n = 270	n = 267		n = 280	n = 275	
Age, years										
≤50	213	95	113		105	98		112	100	
>50	349	182	161	0.093	165	169	0.602	168	175	0.378
Tumor size median										
≤22 mm	283	144	132		137	129		140	138	
>22 mm	278	132	142	0.348	132	138	0.545	139	137	1.000
N.A.	1									
Nodal status										
pN0	65	27	37		28	33		37	28	
pN+	497	250	237	0.169	242	234	0.468	243	247	0.267
Histological type										
Ductal	399	181	211		171	213		204	192	
Lobular	110	66	43		69	33		47	60	
Other	53	30	20	0.010	30	21	<0.001	29	23	0.274
Histological grade										
Grade 1	46	33	13		26	16		20	25	
Grade 2	264	138	119		153	97		127	132	
Grade 3	250	106	140	0.001	91	152	<0.001	131	118	0.518
N.A.	2									
ER status										
Positive	405	215	181		214	171		191	207	
Negative	157	62	93	0.003	56	96	<0.001	89	68	0.065
HER-2 status										
Positive	170	85	83		72	93		93	77	
Negative	392	192	191	0.920	198	174	0.040	187	198	0.183
Biological group										
ER+, HER-2−	314	165	142		173	123		139	168	
ER+, HER2+	91	50	39		41	48		52	39	
ER-, HER2+	79	35	44		31	45		41	38	
ER-, HER2−	78	27	49	0.015	25	51	<0.001	48	30	0.032
Ki67										
≤20 %	271	149	116		158	96		124	140	
>20 %	242	104	134	0.005	92	144	<0.001	126	116	0.252
N.A.	49									

Results are presented as number of patients. ^a^The cutoff values used correspond to the median values of number of positive cells in the entire series. ^b^Chi-square test. *ER* estrogen receptor, *HER-2* human epidermal growth factor receptor 2, *N.A.* not available

### Immunohistochemical analysis

Sections (4-μm) of the TMA blocks were stained using standard immunohistochemical techniques for the expression of CD68 (anti-SA2 antibody clone 3C6, Abcam, Cambridge, UK), CD163 (clone 10D6, Novocastra, Newcastle, UK) and HLA-DRα (Dako, Glostrup, Denmark) [30, 31] (detailed information provided in Additional file 1). All the stained TMA slides were scanned using an Olympus virtual microscope equipped with Dotslide using the 10× objective (Olympus BX51, Olympus, Munich, Germany), and AxioCam camera (Zeiss, Jena, Germany). Positively stained cells were counted using Fiji equipment version 1.48s (Wayne Rasband, NIH). After color deconvolution for hematoxylin and 3,3’-Diaminobenzidine (DAB), the threshold was set for macrophage visualization. The size limit for particle analysis was carefully chosen to include only macrophages. Damaged samples were excluded from the analysis. The data were analyzed in a double-blinded fashion. The investigators were blinded to the identity and clinical pathological characteristics of each sample while analyzing/scoring the macrophage content and differentiation status. The final numbers of positive cells per marker, per sample were passed on to hypothesis-naïve investigators who performed the statistical analysis of the cohort.

### Cell culture

Human breast cancer cell lines MCF-7, MDA-MB231 and T47D, obtained from American Type Culture Collection (ATCC), were grown in Roswell Park Memorial Institure (RPMI)-1640 medium (Sigma-Aldrich, St Louis, MO, USA) supplemented with 10 % fetal bovine serum (FBS) (Gibco, Grand Island, NY, USA) and 100 IU/ml penicillin and streptomycin (Gibco, Bleiswijk, Netherlands) at 37 °C in a 5 % CO_2_ atmosphere. After reaching confluence, cell culture medium was changed to medium containing only 1 % FBS and kept in culture for 72 h. At the end of the culture period, the CM were collected from at least three independent cell line batches from each cell type. The CM were centrifuged for 5 minutes at 2,800 g, aliquoted and frozen at −20 °C. CM were used as 50 % supplement of the macrophage differentiation culture medium together with 10 % FBS. The cell lines were recently authenticated by STR (Short tandem repeat) profiling by a certified cell line authentication service (DDC Medical, Fisher Scientific, London, UK). Mycoplasma detection was performed on a routine basis by 4’,6-Diamidino-2-phenylindole (DAPI) staining of cultured cells.

### Peripheral blood mononuclear cell (PBMC) isolation

PBMCs from five different donors were isolated by centrifugation over Ficoll gradient (Sigma-Aldrich, St Louis, MO, USA). CD14^+^ cells were magnetically labeled with α-CD14 microbeads and positively selected by MACS technology (Miltenyi Biotec, Cologne, Germany).

### Macrophage differentiation

To obtain M1, M2a and M2c macrophages, CD14^+^ monocytes were cultured in MEM (Lonza, Basel, Switzerland) supplemented with 10 % FBS (Gibco, Grand Island, NY, USA) (control, CTR), with IFN-γ (50 ng/ml; M1), or IL-4 (50 ng/ml; M2a), or IL-10 (50 ng/ml; M2c) for 5 days with replacement of half of the culture media at day 3 [32]. To assess the effect of breast cancer cell-line-secreted factors, the same differentiation protocol was carried out in the presence or absence of 50 % CM from MDA-MB231, MCF-7 or T47D cells. For activation status experiments (ELISA), cells were treated with LPS (10 ng/ml, Sigma-Aldrich, St Louis, MO, USA) for one additional day. Unless otherwise stated, all the used cytokines were from R&D Systems (Minneapolis, MN, USA). Supernatants were collected, centrifuged for 5 minutes at 2,800 g, aliquoted and stored at −20 °C until further analysis. Cells were harvested with Accutase (Invitrogen, Paisley, UK), debris were removed by centrifugation (5 minutes at 400 g), and cells were used for flow cytometry analysis. Supernatants were used for ELISA and zymography.

### Flow cytometry

Ex vivo polarized macrophages were analyzed by validated flow cytometry methods [5], with the BD LSR II flow cytometer (BD Biosciences, Erembodegem-Dorp, Belgium). In brief, cells were washed with PBS 0.1 % BSA (Sigma-Aldrich, St Louis, MO, USA) and before staining, Fc receptors were blocked with FcR blocking reagent (BD Biosciences, Erembodegem-Dorp, Belgium): 0.2 × 10^6^ cells were incubated with adequate antibody mixes and washed prior to analysis. Surface-marker expression was analyzed with flow cytometry using the following fluorochrome-labeled monoclonal antibodies: CD14-APC-Cy7 (clone61D3; eBioscience, Paris, France), CD16-PE-Cy7 (clone DJ130c; AbD Serotec, Kidlington, UK), CD64-AF488 (clone 10.1; BioLegend, San Diego, CA, USA), CD200R-PE (clone OX108; AbD Serotec, Kidlington, UK), CD163-AF647 (clone GHI/61; BD Pharmingen, Erembodegem-Dorp, Belgium), and CD86-AF488 (clone IT2.2, BD Pharmingen, Erembodegem-Dorp, Belgium). Equivalent amounts of isotype-matched control antibodies and unstained cells were included in all experiments as negative and autofluorescence controls. Data were analyzed with BD FACSDiva software, after gating on the myeloid population in the FSC/SSC plot. Values were expressed as the percent ratio of the median fluorescence intensity (MedFI) of the marker of interest over the MedFI of the unstained cells.

### ELISA

LPS-activated macrophage culture supernatants were used in ELISA for quantification of h-IL-10, h-IL-8, and h-IL-6 according to the manufacturer’s instructions (R&D systems). h-M-CSF was quantified in breast cancer cell line CM (Duo set, R&D systems, Minneapolis, MN, USA).

### Zymography

The potential proteolytic activity of MMPs in the supernatants of the obtained macrophages was determined by zymography as previously described [33]. The stained polyacrylamide-gelatin gels were observed with the Image Quant RT ECL imager. Densitometry of the bands corresponding to pro-MMP-9 activity (92 kDa) was performed using Fiji equipment version 1.48s (Wayne Rasband, NIH). Presented values are the optical densities of pro-MMP-9-digested bands normalized to the total protein content of the corresponding total cell lysate compared with the density of the equivalent background area.

### Statistics

TMA results were analyzed with SAS version 8.2 for Windows (SAS Institute, Cary, NC, USA) using the median values of the numbers of positive cells in the entire series as the cutoff value. Frequency tables were analyzed using the chi-square (χ^*2*^) test. Survival between groups was compared using the Kaplan-Meier life-table method and a Cox multivariate proportional hazards model. The log-rank test was used to confirm the robustness of the analysis. The subgroup analyses were performed including the macrophage markers, the subgroup variable, and their interaction in the Cox model. The Mann-Whitney or Kruskal-Wallis tests were applied when suitable. All *P* values are two-sided and are not adjusted for multiple testing. Experimental data were expressed as median ± SD, unless otherwise indicated. The Kruskal-Wallis test followed by Dunn’s post hoc test was employed to calculate statistically significant differences between the CTR and the various conditions, using GraphPad Prism software.

### Study approval

Permission to use the tissues from the FinXX study for research purposes was provided by the Finnish Ministry of Social Affairs and Health. The ethics committee at the Helsinki University Central Hospital (Helsinki, Finland) approved the FinXX study and the current study (permission HUS 35/13/03/02/2015). Ethical approval for the use of peripheral blood from healthy donors was obtained from the Nantes University Hospital Ethics Committee. Samples were obtained from the Établissement Français du Sang with informed consent (agreement reference NTS 2000–24, Avenant n°10).

### Online supplemental material

A supplemental table (Additional file 1) and supplemental figures (Additional files 2, 3, 4, 5 and 6) are available online.

## Results

### Clinical significance of TAM numbers and differentiation status in breast cancer patients

**Fig. 1:**
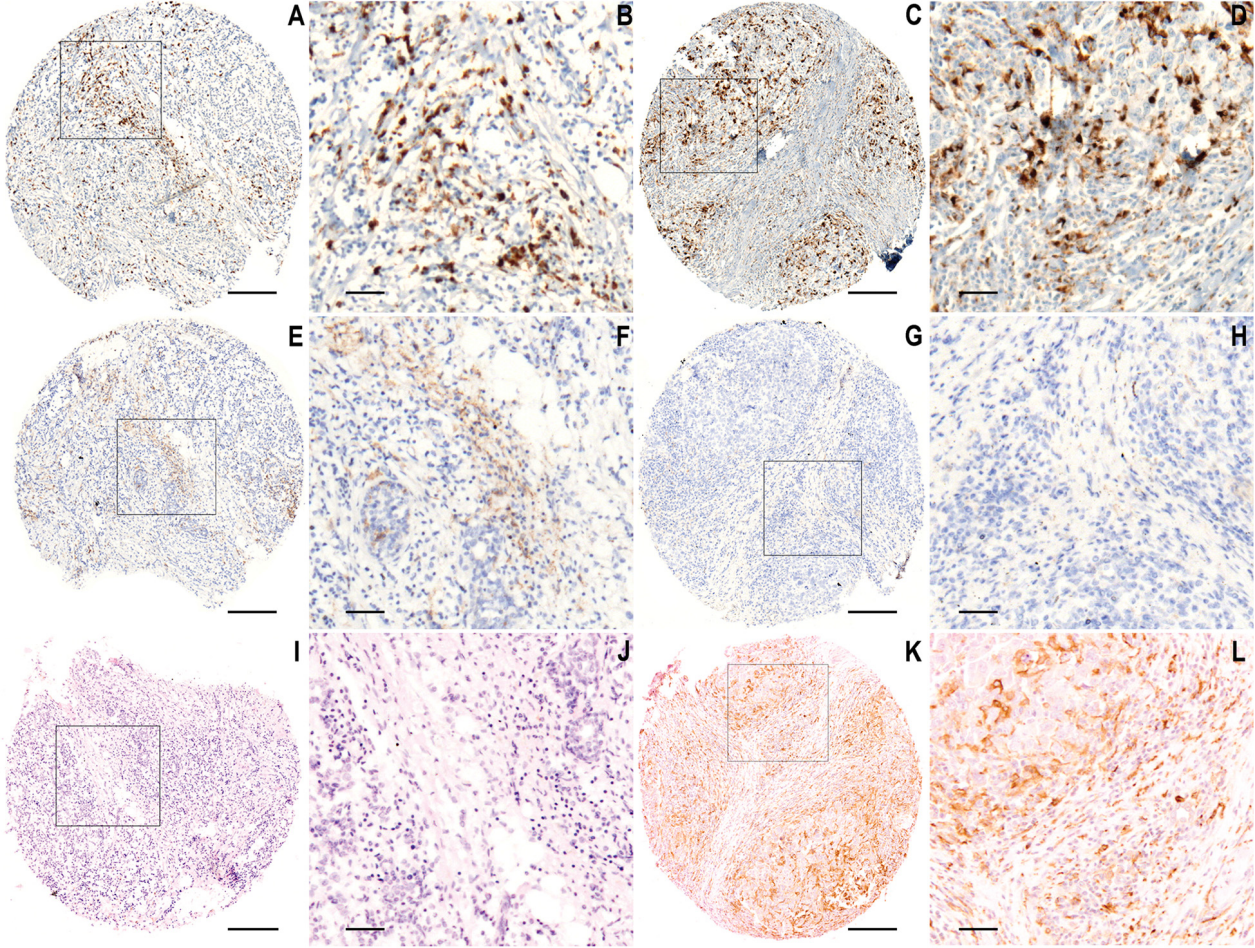
Representative images of tissue microarray (TMA) staining revealing interpatient heterogeneous macrophage marker expression levels. CD68 (**a**-**d**), HLA-DRIIα (**e**-**h**) and CD163 (**i**-**l**). Patient core overview (**a**, **c**, **e**, **g**, **i**, **k,** scale bars 200 μm) and a detailed view of selected area (**b**, **d**, **f**, **h**, **j**, **l**, scale bars 50 μm). Objective amplification × 10

To explore the clinical relevance of TAM differentiation in breast cancer patients, we evaluated total TAM number (CD68), M1 TAM (HLA-DRα) and M2 TAM (CD163) in a large human breast cancer TMA cohort. There was heterogeneity among the patients in the expression levels of the different macrophage markers (Fig. 1). M2 macrophage number, identified as the umber of CD163^+^ cells, was strongly associated with fast proliferation (Ki67 positivity >20 %), poor differentiation (grade 3), ER negativity and histological ductal type (Table 2). None of the individual markers (CD68, HLA-DRα or CD163) was on its own strongly correlated with prognosis, recurrence-free survival (RFS) or overall survival (Additional file 2). In the multivariate Cox model for RFS, markers such as Ki67 positivity >20 %, node positivity and primary tumor size >22 mm were strongly significant predictive factors (*p* <0.001 for tumor size and <0.01 for the other factors). In the same model CD163 was a significant factor (*p* = 0.011) together with other model covariates, such as ER negativity (Table 3).

**Table 2:** CD68^+^ and CD163^+^ cell number median values and relevant clinical characteristics

Factor	N = 551	CD68^+^ median (range)		*P* ^a^	N = 537	CD163^+^ median (range)		*P* ^a^
Histological type								
Ductal	392	418	(0–3634)		384	208	(2–1772)	
Lobular	109	249	(1–3113)		102	106	(3–802)	
Other	50	265	(0–1397)	<0.001	51	127	(4–1359)	<0.001
Histological grade								
Grade 1	46	240	(18–3113)		42	125	(5–524)	
Grade 2	257	337	(0–3634)		250	115	(2–1229)	
Grade 3	246	436	(0–2595)	<0.001	243	265	(3–1772)	<0.001
N.A.	2				2			
ER status								
Positive	396	340	(0–3634)		385	131	(2–1772)	
Negative	155	435	(0–2595)	0.006	152	268	(6–1743)	<0.001
HER-2 status								
Positive	168	363	(9–2673)		165	221	(2–1772)	
Negative	383	369	(0–3634)	0.900	372	148	(3–1359)	0.002
Biological group								
ER+, HER-2−	307	334	(0–3634)		296	124	(3–1359)	
ER+, HER-2+	89	343	(12–2673)		89	200	(2–1772)	
ER-, HER-2+	79	424	(9–1306.5)		76	227	(7–1743)	
ER-, HER-2−	76	484	(0–2595)	0.023	76	289	(6–1111)	<0.001
Ki67								
≤20 %	265	318	(0–3634)		254	115	(2–1772)	
>20 %	238	431	(0–2794)	0.002	236	263	(3–1743)	<0.001
N.A.	48				47			

^a^Mann-Whitney or Kruskal-Wallis test. *ER* estrogen receptor, *HER-2* human epidermal growth factor receptor 2, *N.A.* not available

**Table 3:** Independent prognostic factors in Cox multivariate model for recurrence-free survival in years

Variables	Regression coefficent	Standard error	Regression coefficient/standard error	*χ* ^2^	*P*	Exp (Coef)	95 % CI	
							Lower	Upper
ER^+^	−0.612	0.252	−2.430	5.903	0.0151	0.542	0.331	0.888
HER-2^+^	0.050	0.239	0.211	0.045	0.8326	1.052	0.659	1.679
Ki67 >20%	−0.711	0.269	−2.642	6.981	0.0082	0.491	0.290	0.832
Node positivity	−1.096	0.394	−2.780	7.730	0.0054	0.334	0.154	0.724
Size >22 mm	−0.865	0.237	−3.657	13.372	0.0003	0.421	0.265	0.669
Histological grade 3	−0.071	0.268	−0.266	0.071	0.7899	0.931	0.550	1.575
CD163 >167.5	0.580	0.229	2.531	6.408	0.0114	1.786	1.140	2.798

*ER* estrogen receptor, *HER-2* human epidermal growth factor receptor 2, *Coef* regression coefficient, *χ* ^2^ chi-squared, *Exp(Coef)* hazard ratio

### Human breast cancer cells condition ex vivo differentiation and activation of human macrophages

**Fig. 2:**
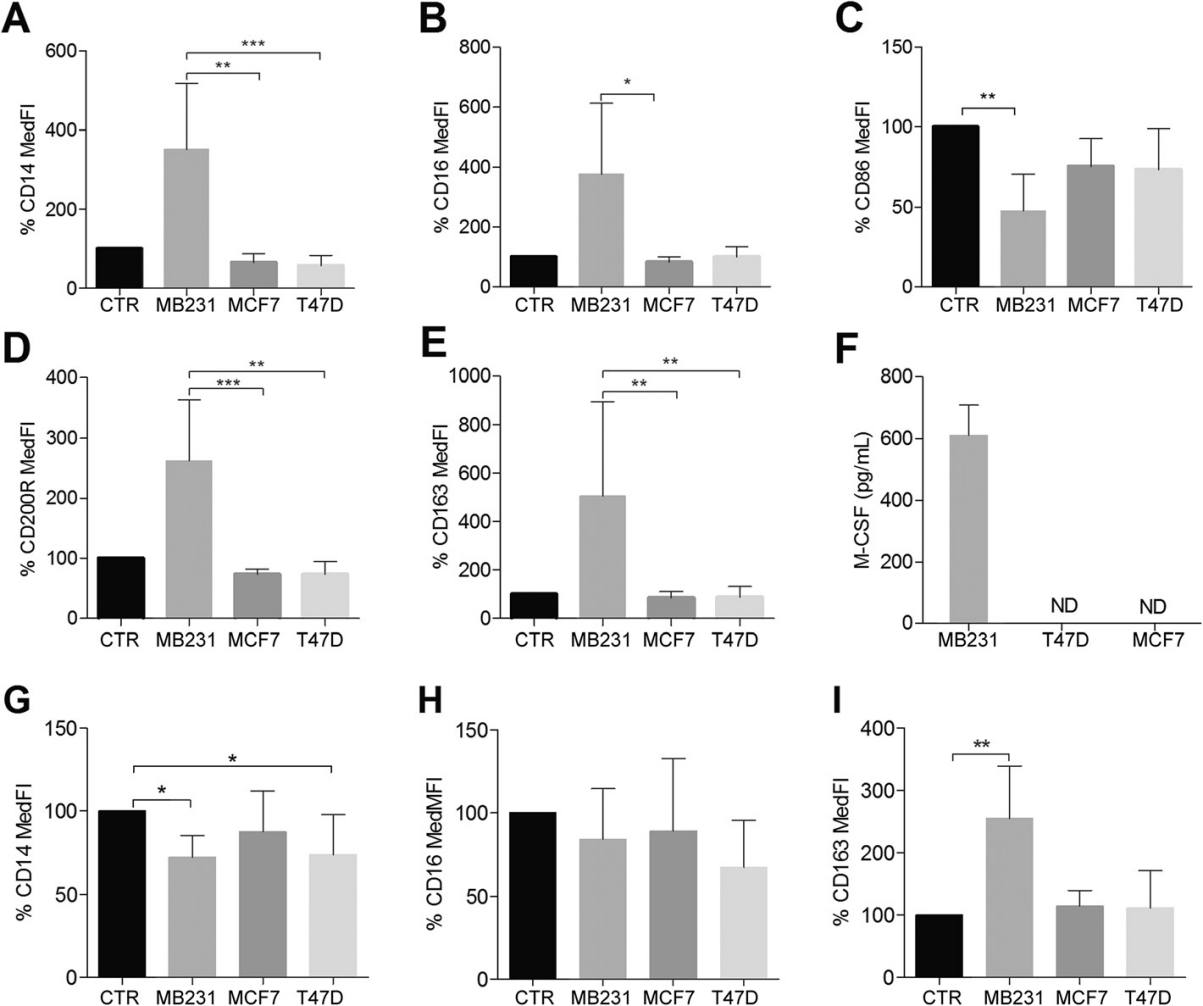
Flow cytometry analysis of CD14^+^ cells differentiated for 5 days with or without 50 % conditioned media (CM). **a**-**e** Percentual variation of median fluorescence intensity (*MedFI*) of CD14, CD16, CD86, CD200R and CD163 compared to control (*CTR*), n = 5. **f** Monocyte colony stimulating factor (*M-CSF*) protein levels in breast cancer cell line CM (n = 3). **g**-**i** Flow cytometry analysis of CD14^+^ cells differentiated in the presence of IL-10 for 5 days with or without 50 % breast cancer cell line CM. Percent variation of MedFI of CD14, CD16 and CD163 compared to CTR (IL-10 alone). *Error bars* represent + SD, n = 5 *p <0.05, **p <0.005 (Kruskal-Wallis analysis followed by Dunn’s post hoc test). *MB231*, MDA-MB231 CM

**Fig. 3:**
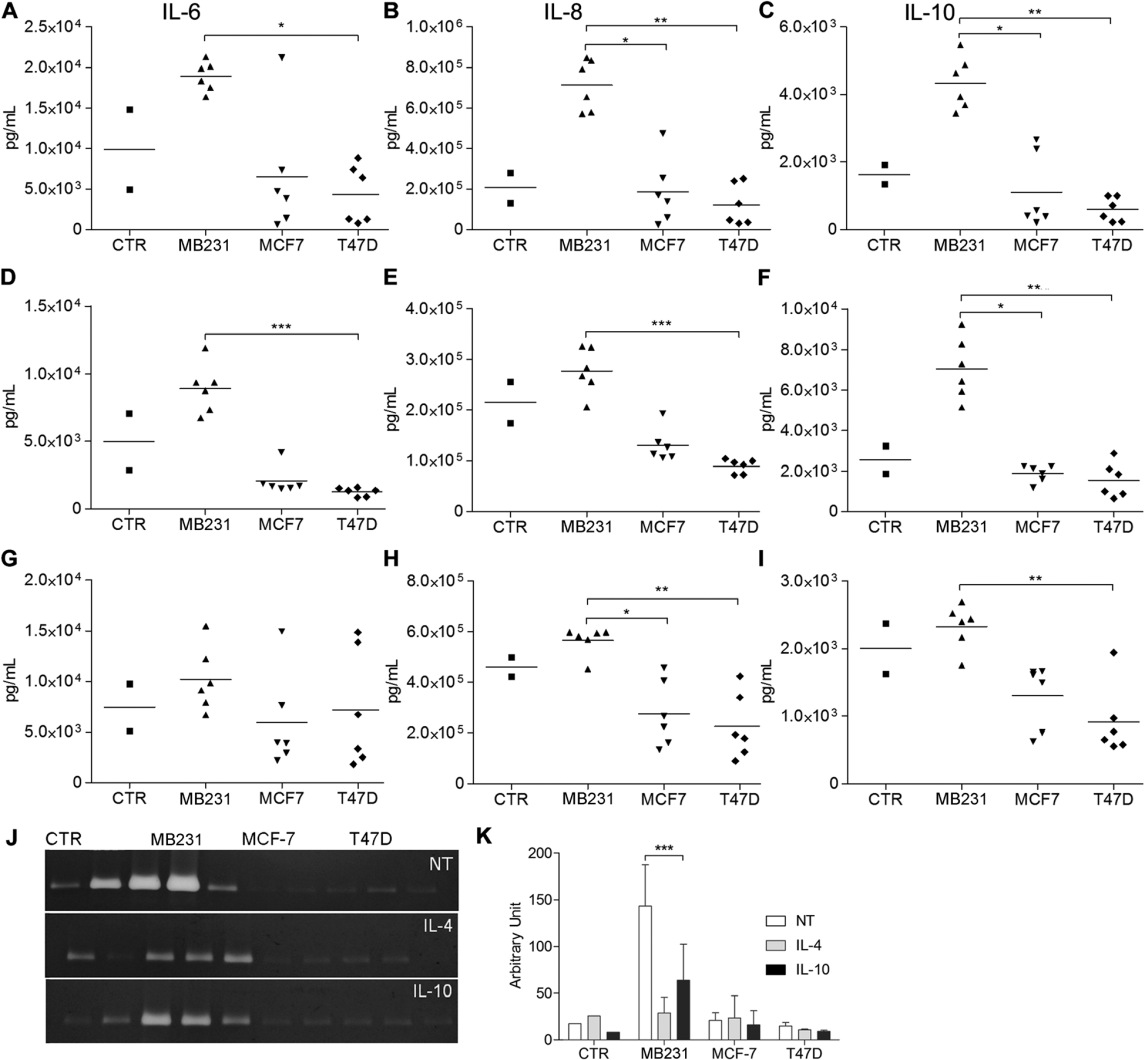
Cytokine secretion profile after 24 h of lipopolysaccharide (LPS)-stimulation. **a**-**c** CD14^+^ cells differentiated for 5 days with or without 50 % breast cancer cell line conditioned media (CM) (**d**-**f)** in the presence of IL-4 (**g**-**i**) in the presence of IL-10. **a**-**i** All results are the mean of three experimental replicates and two biological replicates. **j** Representative matrix metalloproteinase (MMP)-9 zymography gel (**k**) relative MMP-9 activity, expressed as digested band optical density normalized to equivalent background area optical density. *Error bars* represent + SD, n = 3, *p <0.05, **p <0.005, ***p <0.0005 (Kruskal-Wallis analysis followed by Dunn’s *post hoc* test). *MB231*, MDA-MB231 CM; NT, non-treated

**Fig. 4:**
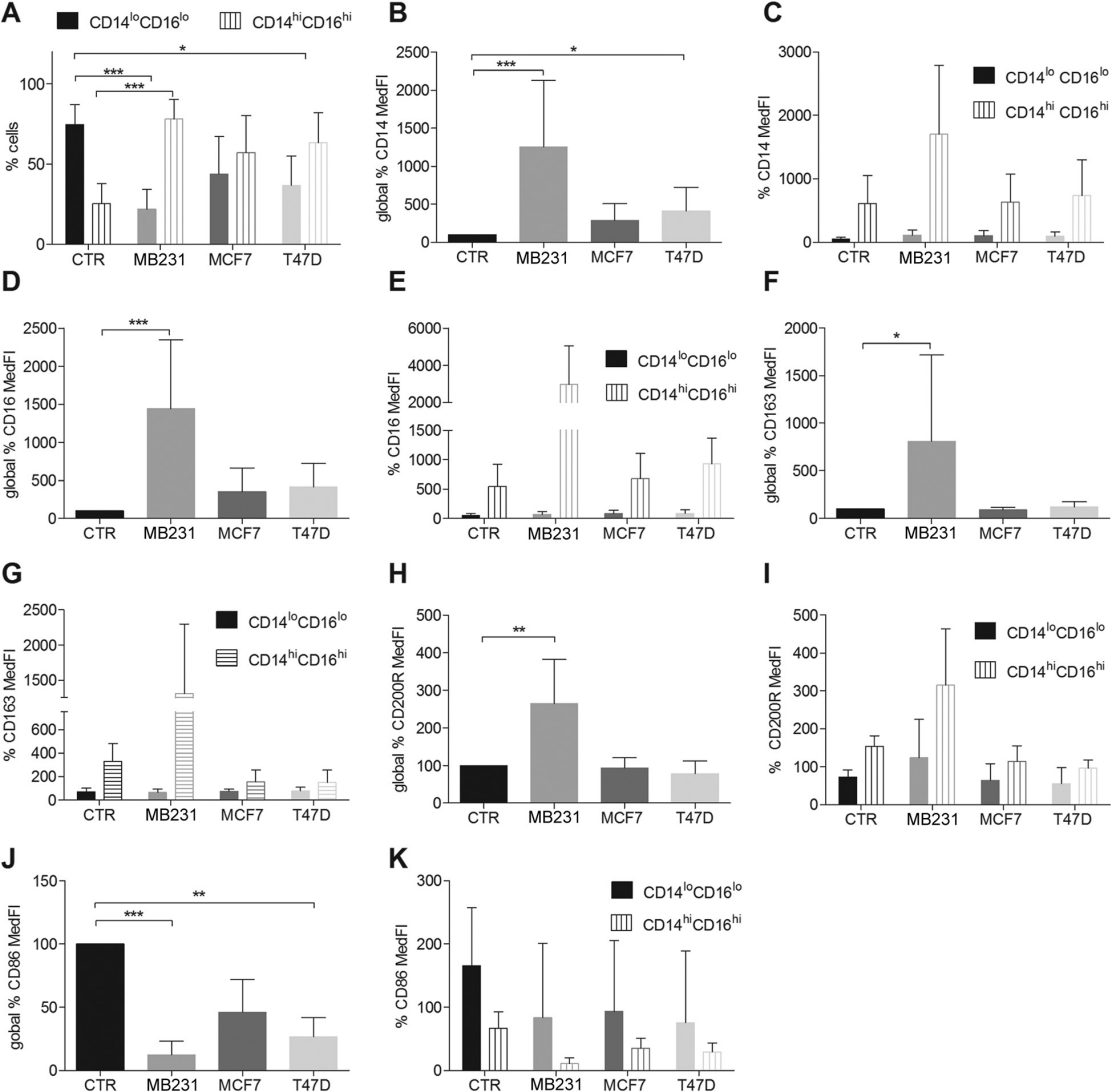
Flow cytometry analysis of CD14^+^ cells differentiated for 5 days in the presence of IL-4 with or without 50 % conditioned media (CM). **a** CD14^lo^CD16^lo^ and CD14^hi^CD16^hi^ subpopulation distribution. **b**, **d**, **f**, **h**, **j** Overall percent variation of CD14, CD16, CD163, CD200R and CD86 median fluorescence intensity (*MedFI*), compared with control (*CTR*) (IL-4 alone) (**c**, **e**, **g**, **i**, **k**) CD14 CD16, CD163, CD200R and CD86 MedFI percent variation in the subpopulations CD14^lo^CD16^lo^ and CD14^hi^CD16^hi^. *Error bars* represent + SD, n = 5, *p <0.05, **p <0.005, ***p <0.0005 (Kruskal-Wallis analysis followed by Dunn’s post hoc test). *MB231*, MDA-MB231 CM

As a proof of concept, we showed that the isolated CD14^+^ cells could be differentiated to M1 (high CD64, high IL-6 secretion), M2a (high CD200R and CD86, low IL-6 and high IL-8 secretion) and M2c macrophages (high CD163, low IL-6 and high IL-10 secretion), respectively, using IFN-γ, IL-4 and IL-10, thus, demonstrating their proven [34] ex vivo plasticity (Figs. 2, 3 and 4 and Additional file 3). Considering M1 differentiation in the presence of IFN-γ, none of the CM affected the expression levels of M1 surface-markers (Additional file 4) nor the secretion profile (data not shown).

CD14^+^ cells differentiated in the presence of MDA-MB231 CM alone yielded an M2 macrophage population (Fig. 2a-e). This result was most obvious in terms of CD86, CD200R and CD163 expression levels (Fig. 2c-e). The differentiation only in the presence of other CM retained the control phenotype features (Fig. 2a-e and Additional file 3).

Macrophages differentiated in the presence of MDA-MB231 CM produced higher amounts of IL-6, IL-8, IL-10 (Fig. 3a-c) and MMP-9 (Fig. 3j-k) than the CTR or other CM. That scenario remained true when the cells were concomitantly treated with IL-4 (Fig. 3d-f) or IL-10 (Fig. 3g-i), except for IL-6 secretion in the latter treatment (Fig. 3g).

### MDA-MB231 cells secrete large amounts of M-CSF, skewing macrophages to an M2c-like phenotype

The strong effects of the MDA-MB231 cells led us to inspect all the CM for dissimilarly secreted macrophage-differentiating factors. We saw no differences in terms of IL-10, transforming growth factor (TGF)-β or IL-4 (data not shown), but only MDA-MB231 cells secreted high amounts of M-CSF (Fig. 2f).

CD14^+^ cells differentiated in the presence of IL-10 and MDA-MB231 or T47D CM (Fig. 2g-i and Additional file 5) showed a decrease in CD14 expression levels when compared to cells differentiated only with IL-10 (CTR, Fig. 2g). MDA-MB231 CM significantly increased CD163 expression levels (Fig. 2i). The macrophages treated with IL-10 and MDA-MB231 CM secreted more IL-8, IL-10 and MMP-9 than the CTR IL-10-treated macrophages (Fig. 3g-k).

### Human breast cancer cells affect M2a macrophage differentiation, rendering macrophages to a mixed M2a/M2c phenotype

When exposed to IL-4, two major macrophage subpopulations arose in all the conditions: CD14^lo^/C16^lo^ and CD14^hi^/CD16^hi^ (Fig. 4). In the presence of breast cancer cell line CM, the relative percentage of each of these subpopulations changed. Instead of a predominance of the CD14^lo^/C16^lo^ population (CTR, Fig. 4a), there was a statistically significant inversion toward a predominance of the CD14^hi^/CD16^hi^ population (Fig. 4a), especially in the presence of MDA-MB231 (*p* <0.0005) or T47D CM (*p* <0.05). MCF-7 CM showed the same trend reaching equilibrium between the two subpopulations not significantly statistically different from the subpopulation distribution in the CTR (IL-4 alone). The detailed analysis of the surface-markers from those subpopulations indicated that MDA-MB231 and T47D CM increase CD14 (Fig. 4b) and decrease CD86 overall expression (Fig. 4j). MDA-MB231 CM increases CD16 (Fig. 4d), CD163 (Fig. 4f) and CD200R expression (Fig. 4h). These fluctuations are mostly due to the CD14^hi^/CD16^hi^ population (Fig. 4c, e, g, i, k) and the results were statistically significant for the global expression (Fig. 4b, d, f, h, j; *p* <0.05). Although not statistically significant, MCF-7 CM induced the same trend as T47D CM (Fig. 4a-k).

Only MDA-MB231 CM affected cytokine and MMP-9 secretion, reflecting the exceptionality of this cell line (Fig. 3d-f, j, k). MDA-MB231 CM in the IL-4 condition increased IL-10 secretion (Fig. 3f), and increased IL-6, IL-8 (Fig. 3d, e) and MMP-9 secretion (Fig. 3j, k). If compared to MDA-MB231 CM alone, IL-4 combined with MDA-MB231 CM decreased the secretion of IL-6, IL-8 and MMP-9 and increased IL-10 secretion (Fig. 3a-c and Fig. 3j, k). Overall, MDA-MB231 CM in the presence of IL-4 produced a macrophage subpopulation with an intermediate/mixed M2a/M2c phenotype (Additional file 6), with an abundant production of the immunosuppressive M2c-inducing cytokine IL-10. These macrophages retain matrix-remodeling properties by secreting MMP-9. The possibility that the MCF-7 or T47D CM-induced CD14^hi^/CD16^hi^ macrophage subpopulations also secrete different levels of cytokines should not be discarded. Those more subtle differences may be masked by the higher titers produced by the MDA-MB231-CM-induced CD14^hi^/CD16^hi^ subpopulations.

## Discussion

### Clinical significance of TAM numbers and differentiation status in breast cancer patients

In this study CD163^+^ cells in primary breast tumor tissue were brought up as a negative prognosis factor for RFS. However, we could not precisely determine which would be the additional interacting factors involved in this effect. It is clear that CD163 correlates with known factors to be associated with a bad prognosis, such as ER negativity, poor differentiation (grade 3) and ductal type (Tables 1 and 2). Previous studies have shown that higher tumor grade [22] and higher Ki67 index are associated with increased CD68^+^ macrophage infiltration in breast tumors [23, 35]. It was suggested that highly proliferative high-grade tumors elicit an active immune response that further supports angiogenesis and tumor growth. Further, these high-grade tumors may secrete higher levels of macrophage-recruiting/modulating cytokines such as M-CSF (ex vivo results, Fig. 2f), IL-10 and/or TGF-β [6], which is in agreement with the high number of CD163^+^ M2-macrophages. A study exploring stromal gene signatures in DCIS and invasive breast cancer found that higher grade ER-negative and PR-negative tumors are associated with macrophage responses [36]. Macrophage infiltration was present early in the tumor progression at the DCIS stage, and the majority of cases remained positive in matched invasive breast cancer cases, accounting for early macrophage recruitment in breast cancer progression [36]. Although widely accepted as a specific monocyte/macrophage marker, CD163 can also be expressed by immature MDCs, which include myeloid-derived suppressor cells (MDSCs), known to favor tumor progression [24]. Therefore, we cannot exclude the possibility that a percentage of the CD163^+^ cells detected may in fact be MDSCs, which could account for the poor prognostic role of CD163.

### Differential ex vivo conditioning of human macrophage differentiation and activation by different breast cancer cell types

Levano et al. [7] explored the cytokine receptor profile of different breast cancer cell types and found that basal-like cells (e.g., MDA-MB231) express preferentially granulocyte monocyte colony stimulating factor (GM-CSF), hepatocyte growth factor receptor (HGFR, also known as c-MET), CD44, epithelial growth factor receptor (EGFR), transforming growth factor receptor 2 (TGFR2) and oncostatin M receptor (OSMR). Luminal-type breast cancer cells (e.g., MCF-7 and T47D) express RET (a proto-oncogene which encodes for a receptor tyrosine kinase for members of the glial cell line-derived neurotrophic factor) [7] and leukemia inhibitory factor (LIF) [37]. This suggests that TAMs have a different influence depending on the tumor subtype, as breast cancer cells will have different receptors for TAM-derived factors [7]. Further, mesenchymal or epithelial-like breast cancer cells respond to or influence TAMs differently. It has been shown that mesenchymal-like breast cancer cells secrete GM-CSF to activate macrophages to a CCL18-expressing TAM-like phenotype and, reciprocally, these TAM-like macrophages sustain the EMT of cancer cells [38]. These findings are not totally unexpected when considering the role of macrophages in mammary gland development during embryogenesis, puberty, pregnancy and lactation. Macrophages in the mammary gland were proven essential in supporting and activating mammary stem cells necessary for normal morphogenesis [39], branching [9] and in the postpartum-related influx of M2 macrophages [10]. All these developmental processes occur via mechanisms similar to molecular cancer mechanisms, such as vascular endothelial growth factor A (VEGF-A)-stimulated angiogenesis. Additionally, TAMs secrete EGF, TNF-α, VEGF and basic fibroblast growth factor (bFGF) and have reduced antigen presenting ability. Also the release of IL-10 by both tumor cells and TAMs immunosuppresses cytotoxic T-lymphocytes (CTLs) [9].

A study of murine and human macrophage polarization profiles showed that M-CSF-differentiated human macrophages are pro-M2, meaning that LPS or IFN-γ stimulation can still induce an M1 response. However, if stimulated with IL-4 or IL-10 they become more M2 type than the basal macrophages [40]. M-CSF induces CD163 expression in macrophages which, when LPS-stimulated, secrete higher levels of IL-12p40, TNF-α and IL-6 [34]. Our MDA-MB231 cells produce copious amounts of M-CSF (Fig. 2f), in levels similar to clinical samples and MDA-MB231 cells, as reported previously [6]. Similarly, the monocyte shift toward M2/CD163^+^ TAMs by increased levels of M-CSF has been seen in other tumor types such as glioma [41], clear cell renal carcinoma [42], ovarian carcinoma [43] and a mouse model of osteosarcoma [44]. In those tumor types, the elevated M-CSF and CD163 expression correlates with higher tumor grade [41, 42].

CD163 is a monocyte/macrophage-restricted scavenger receptor. It clears hemoglobin/haptoglobin complexes, hence protecting tissues from hemoglobin-induced oxidative damage [45]. It was recently shown that breast cancer CD163^+^ TAMs correlate with Wnt5a expression, the latter factor being responsible for macrophage reprogramming to an anti-inflammatory M2 status. The same group has reported that Wnt5a acts as a feedback antagonist of toll-like receptor (TLR) signaling, inducing IL-10 secretion [26]. Our results fit this mechanism well, as MDA-MB231 CM induced CD163 expression, a feature of M2c TAMs. This could indicate a parallel increase in Wnt5a that inhibits TLR response and increases IL-10 secretion upon LPS stimulation (Fig. 3c, f, i).

As previously discussed, the M2c-boosted differentiation by MDA-MB231-secreted products may have consequences in terms of microenvironment-aided tumor progression via immunosuppressive, matrix remodeling and scavenging TAM functions. These effects may impair an effective immune tumor rejection as our in vitro findings with murine macrophages and breast cancer cell line CM indicate [46].

The overall decrease of CD86 expression in M2a macrophages by breast cancer CM may contribute to immunosuppressive, tumor-promoting behavior. CD86, also known as B7-2, is a type I transmembrane protein of the immunoglobulin superfamily. It is a co-stimulatory molecule expressed by antigen-presenting cells such as dendritic cells and macrophages. Its binding to CD28 on naïve T-cells is essential for Th2 differentiation, cytokine secretion and induction of effector function [47].

In our system, M2a macrophages differentiated in the presence of IL-4 and MDA-MB231 CM had the potential for increased CD200R signaling which in vivo would indicate an immunosuppressive tumor-promoting environment. CD200R is a myeloid receptor expressed on macrophages, granulocytes, dendritic cells and NK (natural killer) cells [48]. CD200R signaling is known to increase the immune activation threshold, being physiologically relevant in restraining inflammation [48]. CD200 ligand interaction with its receptor CD200R on macrophages decreases TNF-α and IFN-γ secretion [49]. CD200 is expressed by cancer cells and other cell types like mesenchymal stem cells, thymocytes, activated T cells, B cells and dendritic cells. Studies in different tumor types, including breast cancer, showed that CD200-CD200R interaction delivers an immunosuppressive signal. This signal directly decreases inflammatory cytokine secretion by macrophages, and indirectly increases regulatory T cells (Treg) and decreases effector T-cell numbers, thereby promoting tumor progression by immune evasion [50].

### Limitations of the study

In the TMA analysis the median of positive cells for each marker in all samples was used as a cutoff value and analysis of normal breast tissue - probably carrying resting macrophages as a healthy baseline control - could not be included. However, our study brought up differences in TAM activation status between patients relevant to the disease outcome. Larger studies are thus justified to ascertain the exact role of M2 macrophages in disease progression.

In the ex vivo macrophage differentiation studies, the extrapolation of the MDA-MB231 CM effects on macrophage differentiation to the clinical situation of triple-negative breast cancer patients should be made with care. MDA-MB231 is an aggressive model cell line, relevant in the field as it is the parental cell line for several metastatic sub-clones widely used in experiments in vivo [51–53]. The MDA-MB231 cell line belongs to the mesenchymal-like subtype, while cancer cells from triple-negative breast tumors, which can be of seven different subtypes, have phenotypic diversity from epithelial to mesenchymal characters [54]. However, we think our study remains relevant as it shows that breast cancer cells, regardless of their hormone receptor status and epithelial/mesenchymal nature, secrete factors that educate macrophages toward M2 differentiation. The most aggressive one, MDA-MB231, did it most effectively. Further studies are needed to unveil the factors responsible for the effects seen. M-CSF appears to be a key factor in M2 TAM differentiation, as shown by others [6, 55], but as breast cancer cell lines (MCF-7 and T47D) that do not produce M-CSF also affected the M2 phenotype, inducing M2a differentiation, we think there are other relevant M2 skewing factors, which our work cannot address. The discovery of such factors is of utmost relevance, and calls for further studies.

## Conclusions

This study combines several lines of evidence for the importance of TAM polarization status in breast cancer progression. For the first time, it is clear that CD163^+^ TAMs associate with other known prognostic factors like fast proliferation, poor differentiation and ER-negativity. CD163^+^ TAMs may be associated with a decrease in RFS according to the multivariate Cox model. The presented ex vivo results are to our knowledge the first demonstrating the modulation of macrophage differentiation solely by breast cancer cell-secreted factors, providing evidence for the mechanisms of breast cancer macrophage education behind clinical findings. Particularly, the mesenchymal-type cell line MDA-MB231 polarizes macrophages toward a mixed M2a/M2c status. It is therefore rational to venture that the screening of TAM activation in breast cancer patients could be useful in predicting patients with a high metastatic risk. The knowledge of TAM activation status may allow the therapeutic targeting of TAMs, once TAMs targeting/modulating agents pass clinical trials and become widely available. These include bisphosphonates [56]; M-CSF and M-CSFR inhibitors and targeting antibodies [57], NCT01316822, NCT01444404; anti-macrophage migration inhibitory factor, NCT01765790 and L-MTP-PE, NCT00631631. There is a scarcity of therapeutic options for patients with triple-negative metastatic breast cancer, and growing resistance to the available options biased by a continuous focus on cancer cell targets, which are by nature genetically unstable and prone to mutations. Approaches such as ours fuel a necessary paradigm change, contributing to the notion that the immunological tumor microenvironment should be taken into account in the development of new multi-target cancer therapies.

## Additional files

Additional file 1:
**Immunohistochemical analysis conditions.** (PDF 273 kb) Additional file 2:
**Tissue microarray (TMA) analysis of recurrence-free survival and overall survival curves related to the macrophage markers A CD68 B CD163 and C HLA-DRIIα.** (PDF 22622 kb) Additional file 3:
**Flow cytometry analysis of CD14** ^**+**^ **cells differentiated for 5 days with or without 50 % conditioned media (CM), IFN-γ, IL-4 and IL-10 A mean fluorescence intensity (MFI) of CD14 B CD16 C CD64 D CD163 E CD86 and F CD200R, normalized to MFI of unstained cells; n = 3, ***** ***p*** **<0.0005.** (PDF 1833 kb) Additional file 4:
**Flow cytometry analysis of CD14** ^**+**^ **cells differentiated for 5 days in the presence of IFN-γ with or without 50 % conditioned media (CM) A mean fluorescence intensity (MFI) of CD14 B CDC16 and C CD64 normalized to MFI of unstained cells; n = 3.** (PDF 2112 kb) Additional file 5:
**Representative flow cytometry dot plot of IL-10 macrophages A human macrophages in the presence of IL-10 with or without breast cancer cell line conditioned media (CM), detected with antibodies against CD14, CD16 and CD163 B mean fluorescence intensity (MFI) of the analyzed surface-markers.** (PDF 18647 kb) Additional file 6:
**Representative flow cytometry dot plot of IL-4 macrophages A human macrophages in the presence of IL-4 with or without breast cancer cell line conditioned media (CM), detected with antibodies against CD14, CD16, CD163, CD200R and CD86 B mean fluorescence intensity (MFI) of the analyzed surface-markers.** (PDF 2715 kb)

## Abbreviations

ATCC: American Type Culture Collection
bFGF: basic fibroblast growth factor
BSA: bovine serum albumin
CM: conditioned media
CTC: circulating tumor cell
CTLs: cytotoxic T-lymphocytes
CTR: control
DAB: 3,3’-Diaminobenzidine
DCIS: ductal carcinoma *in situ*
DTC: disseminating tumor cell
EGF: epidermal growth factor
EGFR: epidermal growth factor receptor
ELISA: enzyme-linked immunosorbent assay
EMT: epithelial to mesenchymal transition
ER: estrogen receptor
FBS: fetal bovine serum
GM-CSF: granulocyte monocyte colony stimulating factor
HER-2: human epidermal growth factor receptor 2
HGFR: hepatocyte growth factor receptor
IFN-γ: interferon γ
kDa: kiloDaltons
LIF: leukemia inhibitory factor
LPS: lipopolysaccharide
M-CSF: monocyte colony stimulating factor
M-CSFR: monocyte colony stimulating factor receptor
MDCs: myeloid-derived cells
MDSCs: myeloid-derived suppressor cells
MedFI: median fluorescence intensity
MMP-9: matrix metalloproteinase 9
MMPs: matrix metalloproteinases
NK: natural killer
OSMR: oncostatin M receptor
PBMCs: peripheral blood mononuclear cells
PBS: phosphate-buffered saline
PR: progesterone receptor
RET: proto-oncogene, which encodes for a receptor tyrosine kinase for members of the glial cell line-derived neurotrophic factor
RFS: recurrence-free survival
RNS: reactive nitrogen species
ROS: reactive oxygen species
TAMs: tumor-associated macrophages
TGFR2: transforming growth factor receptor 2
TGF-β: transforming growth factor β
TLR: toll-like receptor
TMA: tissue microarray
TNF-α: tumor necrosis factor α
Treg: regulatory T-cells
VCAM1: vascular cell adhesion molecule 1
VEGF-A: vascular endothelial growth factor A
