# Supplementary material for: Human breast cancer cells educate macrophages toward the M2 activation status
Source: Breast Cancer Res. 2015 Aug 5;17(1):101. doi: 10.1186/s13058-015-0621-0 (PMC4531540; doi:10.1186/s13058-015-0621-0)
Supplement: Additional file 1: — Immunohistochemical analysis conditions. (PDF 273 kb) [file 13058_2015_621_MOESM1_ESM.pdf]

Additional file 1. Immunohistochemistry conditions

Primary antibody	Antigen retrieval conditions	Signal Amplification system	Development
CD68 (anti-SA2 antibody clone 3C6, Abcam)	Automated immunostaining device BenchMark XT (Roche Diagnostics/Ventana Medical Systems)		OptiView DAB IHC Detection Kit (Roche Diagnostics/Ventana Medical Systems)
CD163 (clone 10D6, Novocastra)	Heat induced epitope retrieval with Tris-EDTA buffer, pH 9	VECTASTAIN® ABC system (Vector labs)	DAB substrate (Dako)
HLA-DRα (Dako)	Heat induced epitope retrieval with Tris-EDTA buffer, pH 9	VECTASTAIN® ABC system (Vector labs)	DAB substrate (Dako)
