# Supplementary figures and images for: Human breast cancer cells educate macrophages toward the M2 activation status
Source: Breast Cancer Res. 2015 Aug 5;17(1):101. doi: 10.1186/s13058-015-0621-0 (PMC4531540; doi:10.1186/s13058-015-0621-0)

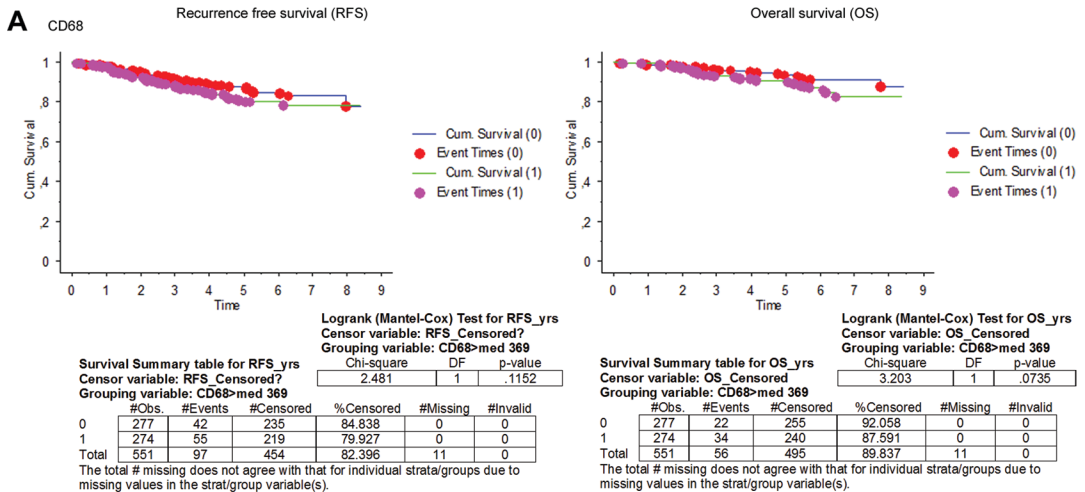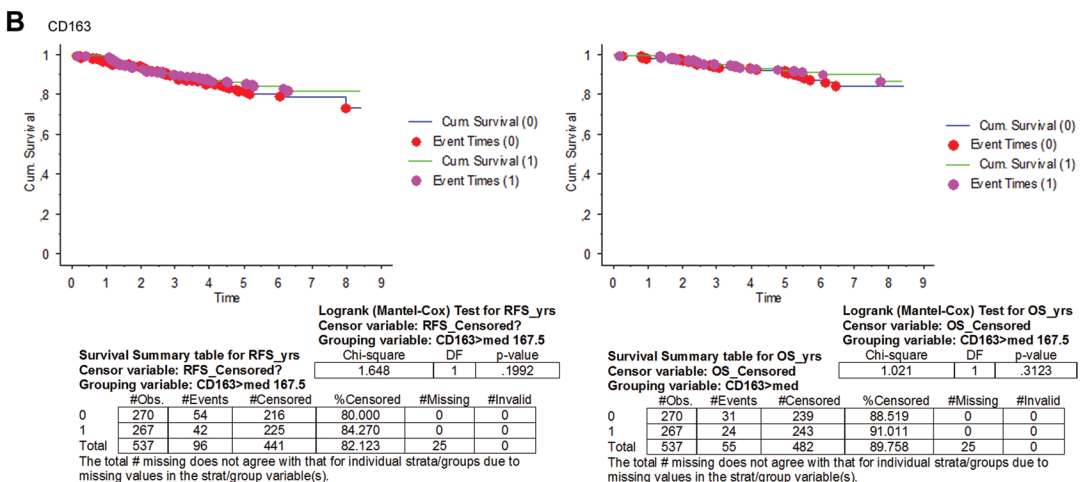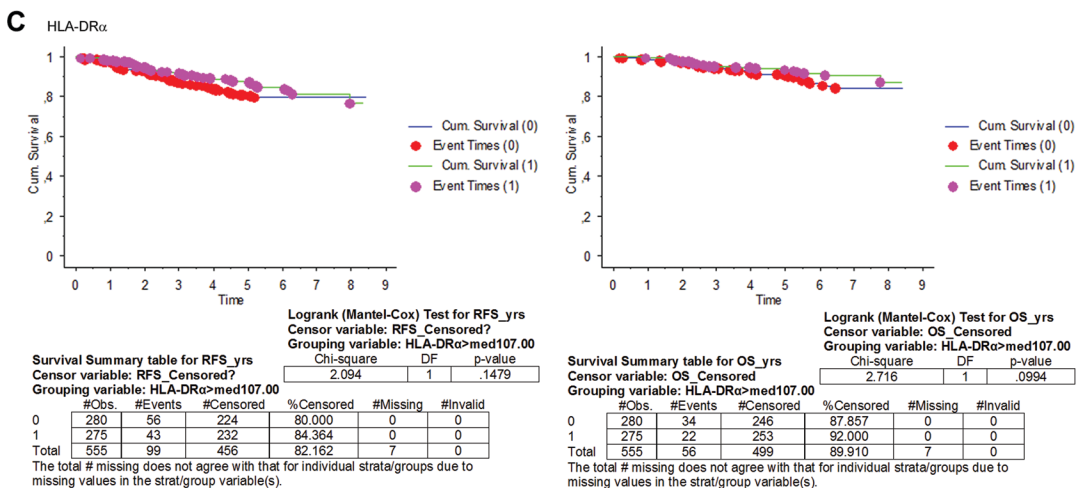

Supplement: Additional file 2: — Tissue microarray (TMA) analysis of recurrence-free survival and overall survival curves related to the macrophage markers A CD68 B CD163 and C HLA-DRIIα. (PDF 22622 kb) [file 13058_2015_621_MOESM2_ESM.pdf]

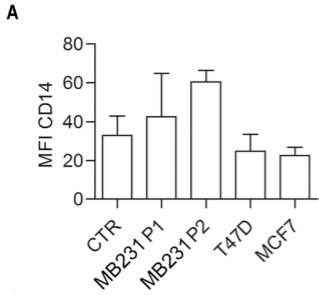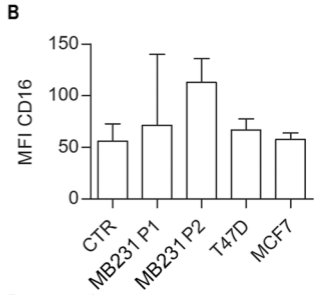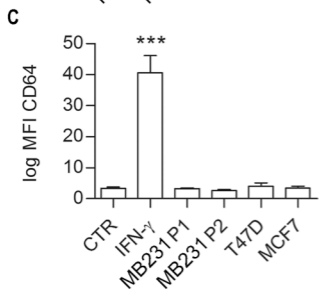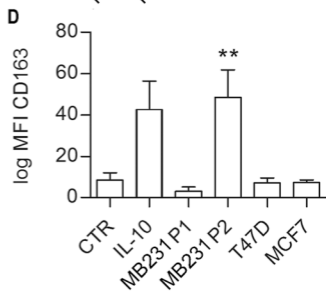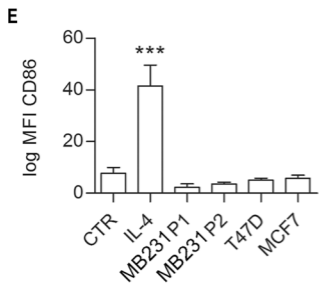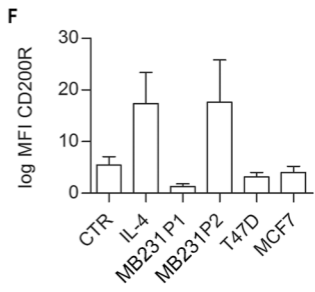

Supplement: Additional file 3: — Flow cytometry analysis of CD14 + cells differentiated for 5 days with or without 50 % conditioned media (CM), IFN-γ, IL-4 and IL-10 A mean fluorescence intensity (MFI) of CD14 B CD16 C CD64 D CD163 E CD86 and F CD200R, normalized to MFI of unstained cells; n = 3, *** p <0.0005. (PDF 1833 kb) [file 13058_2015_621_MOESM3_ESM.pdf]

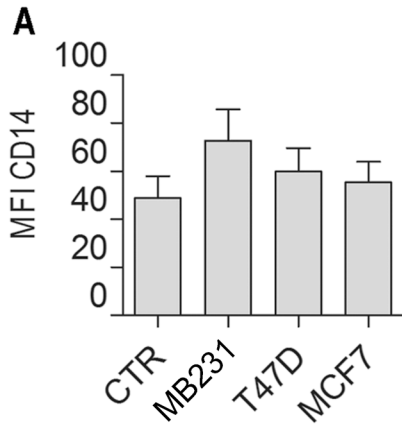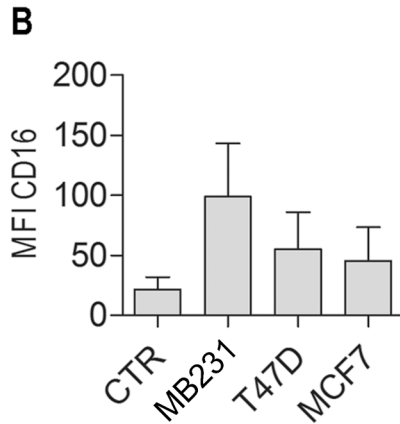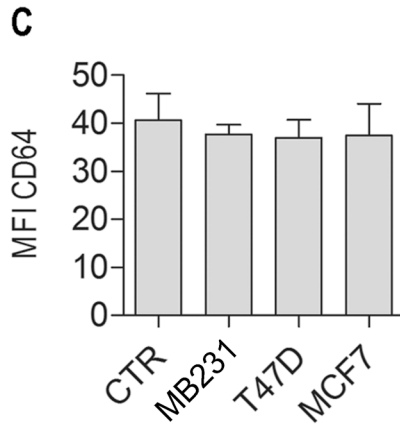

Supplement: Additional file 4: — Flow cytometry analysis of CD14 + cells differentiated for 5 days in the presence of IFN-γ with or without 50 % conditioned media (CM) A mean fluorescence intensity (MFI) of CD14 B CDC16 and C CD64 normalized to MFI of unstained cells; n = 3. (PDF 2112 kb) [file 13058_2015_621_MOESM4_ESM.pdf]

**A**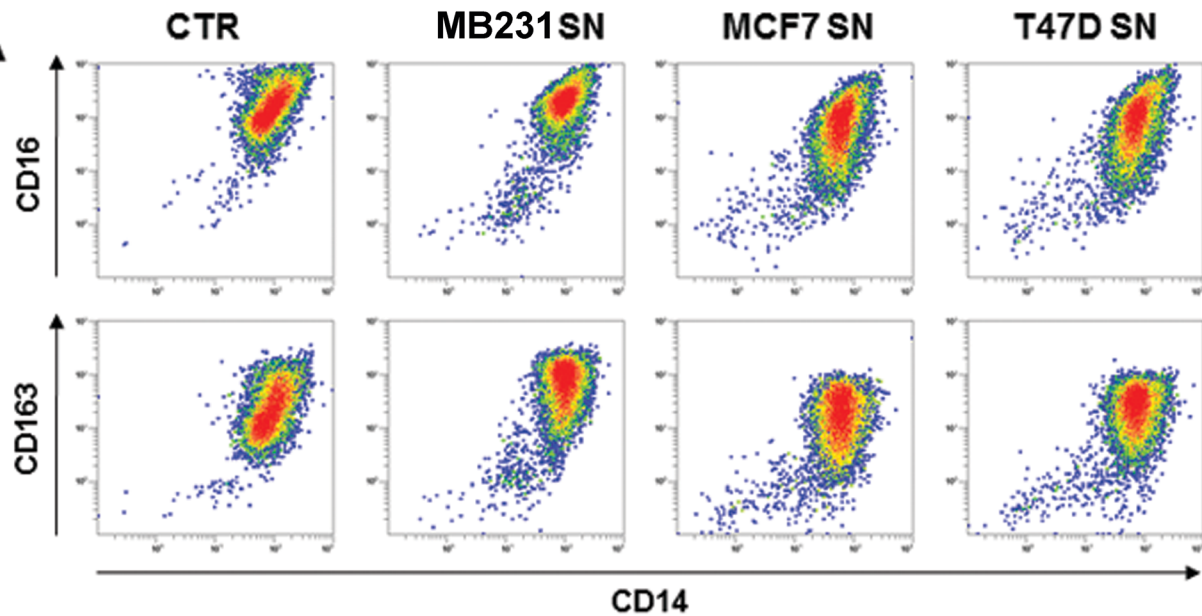**B**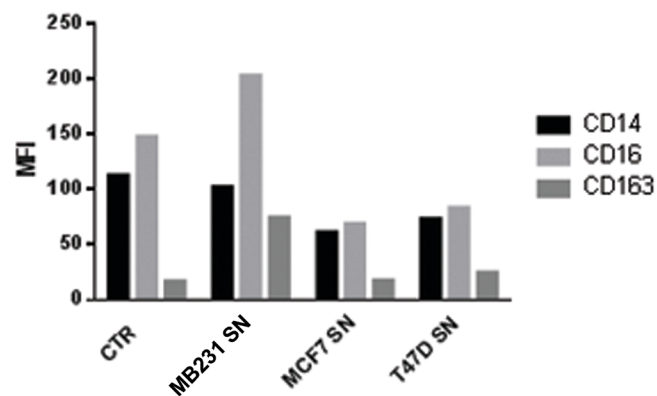

Supplement: Additional file 5: — Representative flow cytometry dot plot of IL-10 macrophages A human macrophages in the presence of IL-10 with or without breast cancer cell line conditioned media (CM), detected with antibodies against CD14, CD16 and CD163 B mean fluorescence intensity (MFI) of the analyzed surface-markers. (PDF 18647 kb) [file 13058_2015_621_MOESM5_ESM.pdf]

**A****CTR****MB231SN****MCF7 SN****T47D SN**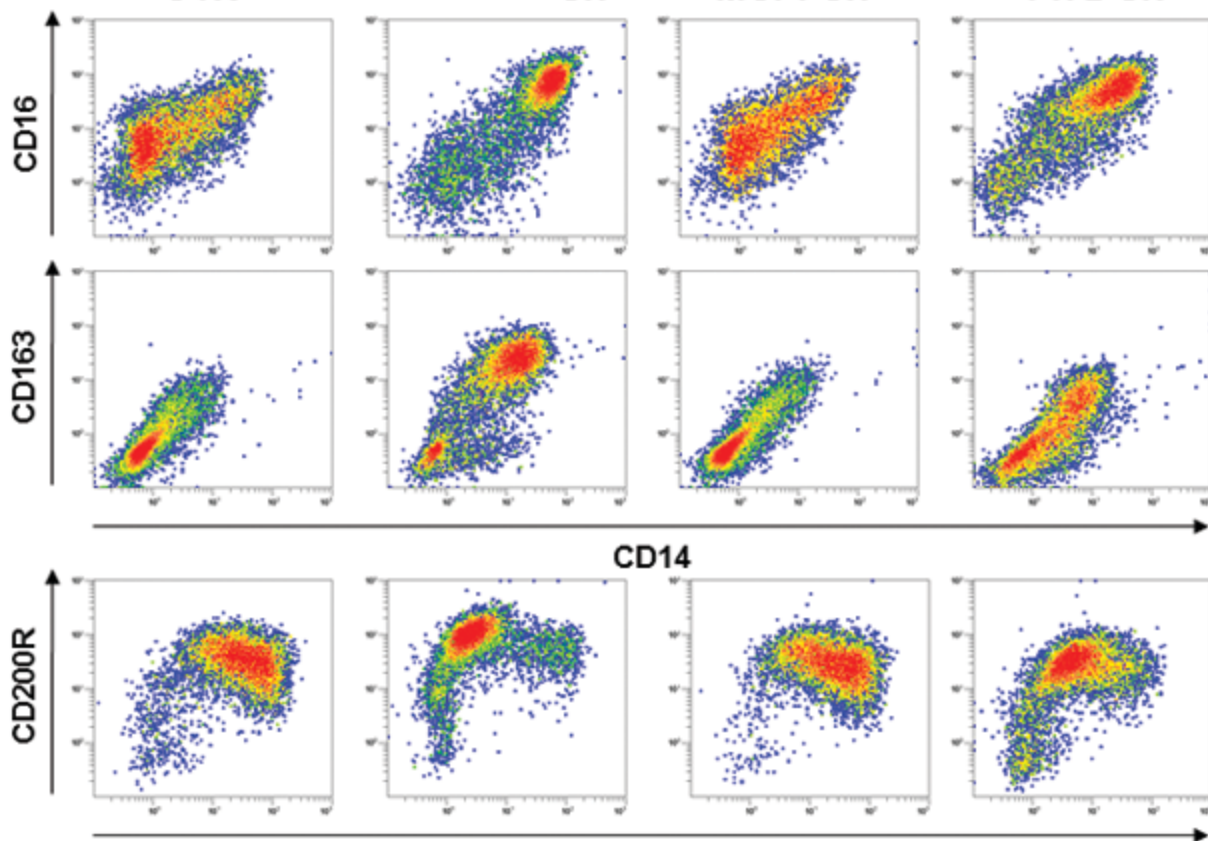**B****CD86**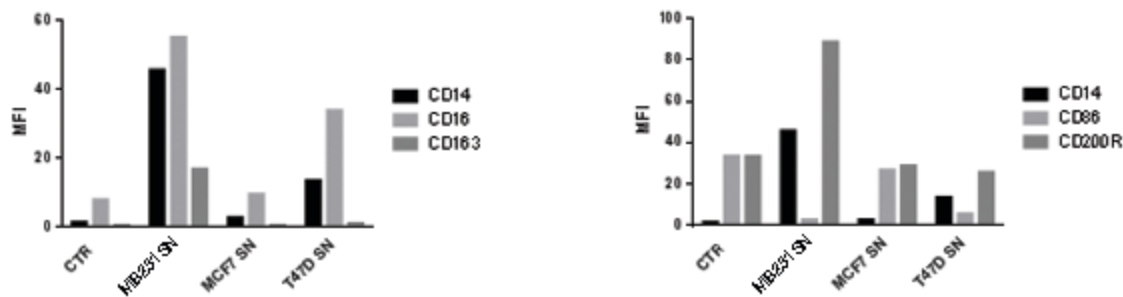

Supplement: Additional file 6: — Representative flow cytometry dot plot of IL-4 macrophages A human macrophages in the presence of IL-4 with or without breast cancer cell line conditioned media (CM), detected with antibodies against CD14, CD16, CD163, CD200R and CD86 B mean fluorescence intensity (MFI) of the analyzed surface-markers. (PDF 2715 kb) [file 13058_2015_621_MOESM6_ESM.pdf]
